# Lipid metabolism dysregulation in solar lentigo: a multi-system-level analysis reveals membrane instability and energy homeostasis disruption

**DOI:** 10.3389/fcell.2026.1751543

**Published:** 2026-03-02

**Authors:** Sohyun Kim, Wonmin Lee, Junghyun Kim, Yoonsung Lee, Kiwon Lee, Man S. Kim, Soon-Hyo Kwon

**Affiliations:** 1 Translational-Transdisciplinary Research Center, Clinical Research Institute, Kyung Hee University Hospital at Gangdong, Kyung Hee University College of Medicine, Seoul, Republic of Korea; 2 Department of Medicine, Kyung Hee University College of Medicine, Seoul, Republic of Korea; 3 Division of Tourism & Wellness, Hankuk University of Foreign Studies, Yongin-si, Gyeonggi-do, Republic of Korea; 4 Department of Bioscience and Biotechnology, Hankuk University of Foreign Studies, Yongin-si, Gyeonggi-do, Republic of Korea; 5 Center for Space Biomedical Science, NEXUS Institute, Kyung Hee University, Yongin-si, Republic of Korea; 6 Department of Dermatology, Kyung Hee University Hospital at Gangdong, Kyung Hee University College of Medicine, Seoul, Republic of Korea

**Keywords:** fatty acid elongation, lipid metabolism, metabolic flux simulation, oxidative stress, solar lentigo, sphingolipid metabolism

## Abstract

Solar lentigo is a common hyperpigmented skin condition caused by chronic ultraviolet exposure, primarily affecting photoaged skin. While previous investigations focused on inflammatory and melanogenic mechanisms, the comprehensive role of lipid metabolism in pathogenesis remains unclear. We aimed to investigate systemic alterations in lipid metabolism and their contribution to solar lentigo development. We performed comprehensive analysis of RNA sequencing data from solar lentigo lesions and control skin samples (n = 7 per group) using metabolic flux simulations, gene co-expression networks, and protein-protein interaction analysis. These multi-system approaches were integrated to identify coordinated alterations in lipid metabolic pathways. Solar lentigo samples exhibited coordinated inhibition of fatty acid elongation, acetyl-CoA carboxylase activity, and sphingolipid biosynthesis, alongside impaired cholesterol synthesis via reduced squalene epoxidase and 7-dehydrocholesterol reductase activity. Compensatory upregulation of phospholipid synthesis enzymes and dihydroceramide desaturases was observed. Pathway disruption and altered calcium signaling, indicating aberrant cellular energy metabolism and membrane integrity. These findings suggest that solar lentigo pathogenesis involves systematic lipid metabolism dysregulation beyond melanogenesis, potentially contributing to membrane instability, energy homeostasis disruption and redox imbalance. The identification of specific metabolic bottlenecks reveals novel targets for lipid-based therapeutic approaches in pigmentary diseases.

## 1 Introduction

Solar lentigo (SL) is a hyperpigmented skin disorder primarily affecting middle-aged and elderly individuals. It manifests as well-defined light-to-dark brown spots commonly located on skin areas with prolonged exposure to ultraviolet (UV) radiation, such as the face. Chronic UV exposure is identified as the main etiological factor in the development of SL (Bastiaens et al., 2004), leading to localized increases in melanin synthesis and alterations in both epidermal and dermal structures (Yonei et al., 2012). Given its strong correlation with photoaging and its impact on aesthetics, significant research efforts have been directed towards understanding the pathophysiological mechanisms underlying SL and exploring effective therapeutic strategies.

Many studies have emphasized the changes in metabolic and gene expression profiles in SL, particularly focusing on alterations in inflammatory gene expression. DNA microarray analyses have revealed the upregulation of genes associated with inflammation in SL lesions, including six genes specifically associated with the inflammatory response, highlighting the significance of microinflammation in photoaged skin (Goyarts et al., 2007). Also, other studies have shown an increased expression of genes involved in both inflammation and fatty acid metabolism in SL (Aoki et al., 2007). These molecular findings offer valuable insights into the pathological features of SL and shed light on the intricate interplay between inflammation and metabolism during SL development.

However, the precise role of lipid metabolism in the SL remains unclear. Although lipid metabolism is recognized as a critical factor in the pathogenesis of various skin conditions (Pietrzak et al., 2010; Kang et al., 2011; Ewald et al., 2015), its involvement in SL has thus far only been suggested indirectly. Studies have reported an increased expression of endothelin-1 and endothelin B receptors in the lesional epidermis (Imokawa, 2019). Although these studies did not explicitly focus on lipid metabolism, endothelin-1 is known to influence lipid metabolism by reducing the uptake of long-chain fatty acids, indicating a potential link between these factors and lipid metabolism in SL (Chien et al., 2011).

Metabolic flux simulations have recently provided innovative insights into disease studies that were previously unexplored (Smith and Robinson, 2011; Batagov et al., 2023). To date, previous studies on SL have mainly focused on analyzing gene or protein expression levels in isolation (Goyarts et al., 2007; Aoki et al., 2007; Motokawa et al., 2005). While investigations into the regulation of inflammation and oxidative stress responses in SL have been conducted, studies utilizing a multi-system-level approach remain relatively limited (Jeong et al., 2024). A recent integrative study by Cai et al. (2025) employed dynamic network driver analysis to identify key regulatory modules in SL, providing a novel perspective on its molecular pathology (Cai et al., 2025). However, comprehensive metabolic flux analysis combined with transcriptomic profiling has not been previously applied to understand the metabolic dysregulation in SL pathogenesis. This study aimed to assess the impact of lipid metabolism in SL using a multi-systemic strategy incorporating differential gene expression patterns, protein-protein interactions, and alterations in metabolic reaction activities.

## 2 Materials and methods

### 2.1 Patient samples and RNA-seq processing

This study utilized SL skin samples that we previously collected from seven patients at Kyung Hee University Hospital at Gangdong, where all procedures were approved by the local Institutional Review Board (IRB). Written informed consent was obtained from all participants prior to sample collection, and the study was conducted in full accordance with the ethical principles for research involving human subjects. Paired skin samples (5 mm diameter) were collected from the SL and adjacent normal skin of seven patients at Kyung Hee University Hospital at Gangdong. The mean age was 82.0 ± 10.2 years (range, 67–96 years). The locations were on the chin (71.4%), cheek (14.3%), and temple (14.3%). Generation of raw sequencing data and subsequent bioinformatic processing were conducted according to our previously published methods (Choi et al., 2025). Key processing steps included read alignment to the human reference genome (GRCh38) and normalization of transcript expression to transcripts per million (TPM) for all downstream analyses.

### 2.2 Expression profiling and pathway enrichment assessment

Differential expression analysis between SL and control samples (n = 7 per group) was performed on the raw count matrix using the DESeq2 package (v1.48.0) in R. Genes were considered significantly differentially expressed if they met a False Discovery Rate (FDR) adjusted p-value <0.05 and an absolute log2 fold change >0.01.

Functional annotation of differentially expressed genes was conducted through Gene Ontology (GO) term enrichment using the clusterProfiler R package (v4.16.0) (Wu et al., 2021). To examine pathway-level changes, Gene Set Enrichment Analysis (GSEA) was applied using pre-ranked gene lists ordered by log2 fold change values, implemented through the fGSEA R package (v1.24.0) (Korotkevich et al., 2016). Metabolic and signaling pathway analysis was performed using KEGG database annotations via the gage package (v2.58.0). Statistical significance for all enrichment tests was set at FDR-adjusted p-value <0.05. Protein interaction networks were constructed by querying the STRING database (v12.0) (Szklarczyk et al., 2023) with the differentially expressed gene set. Network topology analysis and visualization were subsequently performed using Cytoscape (v3.10.3) (Shannon et al., 2003). Functional annotation enrichment analysis was conducted for each PPI network using Gene Ontology biological process terms to elucidate pathway-specific molecular functions.

### 2.3 Clustering analysis

To identify stable gene clusters based on co-expression patterns, consensus clustering was performed on the subset of genes related to the predefined metabolic pathways using the ConsensusClusterPlus (v1.64.0) package in R. The log2 fold change values for these genes were used as the input data matrix. Prior to clustering, the data was centered by the median of each gene. The k-means algorithm with Euclidean distance was applied for clustering. The analysis was repeated 30 times, and the number of clusters (k) was evaluated across a range from 2 to 10 to ensure the stability of the clustering results. The consensus matrix, generated from this analysis, served as a measure of co-expression similarity between all gene pairs.

Genes associated with energy metabolism were selected based on the model’s gene-reaction annotation framework, followed by differential expression analysis using the DESeq2 R package (v1.48.0). Expression pattern-based clustering of differentially expressed genes was executed using the ConsensusClusterPlus R package. The K-means clustering algorithm implemented in ConsensusClusterPlus facilitated the identification of distinct gene expression clusters and enabled the characterization of potential molecular subtypes within the dataset.

### 2.4 Metabolic flux analysis

To simulate cellular metabolism alterations, we employed the custom, constraint-based flux simulation previously developed by our group using the Recon1 genome-scale model (da Silveira et al., 2020; Guarnieri et al., 2023; Duarte et al., 2007). The simulation was governed by two primary constraints: first, corresponding enzyme expression levels were used to set the bounds for reaction fluxes, and second, main energy-associated pathways were set to maximal optimization (specifically, Citric Acid Cycle, Oxidative Phosphorylation, CoA Synthesis, CoA Catabolism, Glycolysis and Gluconeogenesis, NAD Metabolism, Fatty Acid Synthesis, Fatty Acid Oxidation, and Biomass and Maintenance Functions). The Van der Waerden (VdW) test was then used to quantify the resulting metabolic alterations between the two conditions. Metabolic flux simulations utilized reaction identifiers from the BiGG Models database (http://bigg.ucsd.edu/).

## 3 Results

### 3.1 Glycerophospholipid dysregulation and membrane composition changes in solar lentigo

**FIGURE 1:**
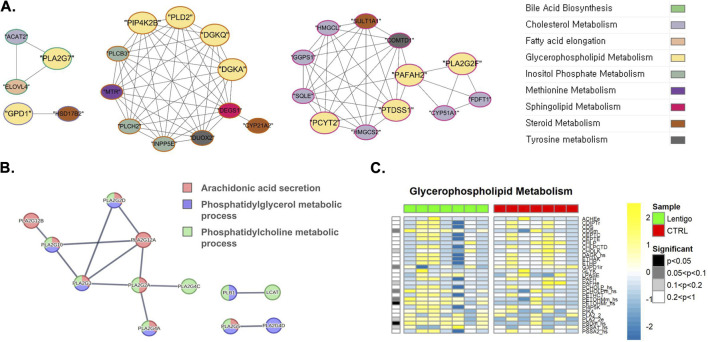
Integrated multi-omics analysis of glycerophospholipid metabolism in solar lentigo (SL). **(A)** Gene co-expression network depicting key glycophospholipid metabolic genes (highlighted in yellow) such as DGKQ, PTDSS1, and PLA2G7, which form distinct co-expression clusters exhibiting strong intra- and inter-pathway connectivity with sphingolipid and methionine metabolism genes. **(B)** Protein-protein interaction (PPI) network showing coordinated upregulation of phospholipase A2 (PLA2) family enzymes (EC:3.1.1.4), indicating enhanced phospholipid turnover and membrane remodeling. **(C)** Metabolic flux simulation demonstrating increased PSDm_hs and PETOHMr_hs reaction activities, representing enhanced conversion of phosphatidylserine (PS) to phosphatidylethanolamine (PE) and phosphatidylcholine (PC), respectively. These alterations suggest concerted transcriptional and metabolic reprogramming of phospholipid synthesis contributing to membrane adaptation under UV stress. Statistical significance determined by Van der Waerden test (FDR-adjusted p < 0.05). Analysis based on RNA-seq data from SL lesions and control skin (n = 7 per group).

In the context of SL, our analysis revealed considerable upregulation of critical phospholipid synthesis reactions. At the transcriptional level, gene co-expression network analysis (Figure 1A) revealed significant regulatory interconnections among key genes involved in glycerophospholipid metabolism. As shown in Figure 1A, these genes (highlighted in yellow), such as *DGKQ, PTDSS1*, and *PLA2G7*, anchored to distinct co-expression clusters that demonstrated strong intra-pathway co-regulation and significant inter-pathway co-expression. Major glycerophospholipid gene clusters centered around DGKQ (closely co-expressed with other glycerophospholipid enzymes such as PLD2) and PTDSS1 (co-expressed with PAFAH2) showed robust connections to genes involved in sphingolipid metabolism (DEGS1) and methionine metabolism. Additionally, *PLA2G7* exhibited co-expression with genes integral to cholesterol metabolism (*ACAT2*) and fatty acid elongation (*ELOVL)*. This intricate network view underscores extensive crosstalk with other critical lipid pathways and supports the observed upregulation of phospholipid metabolic processes in the SL.

Supporting these transcriptional findings, protein-protein interaction (PPI) analysis (Figure 1B; Supplementary Figure S1) demonstrated functional clustering among PLA2 family enzymes (EC:3.1.1.4), including PLA2G10, PLA2G2D, PLA2G3, PLA2G2A, PLA2G4C, PLA2G4A, and PLA2G5, which facilitate arachidonic acid release from phosphatidylcholine. These protein-protein interaction patterns reinforce the coordinated regulation of phospholipid metabolic machinery.

At the metabolic level, flux simulations of the glycerophospholipid pathway (Figure 1C) demonstrated these transcriptional changes translate into functional metabolic alterations. We observed a pronounced increase in Phosphatidylserine decarboxylase reaction (BiGG ID: PSDm_hs) activity, which converts phosphatidylserine (PS) into phosphatidylethanolamine (PE). PE serves as an essential phospholipid that contributes to membrane integrity and stabilization of mitochondrial proteins. Furthermore, substantial upregulation of the PETOHMr_hs reaction was detected, catalyzed by phosphatidylethanolamine N-methyltransferase. This process involves the methylation of phosphatidylethanolamine to produce phosphatidylcholine (PC), utilizing S-adenosylmethionine as a methyl donor and yielding S-adenosylhomocysteine as a byproduct. Given that phosphatidylcholine is a predominant membrane component vital for lipid homeostasis and signaling, its increased synthesis in the SL suggests concerted modifications in membrane phospholipid composition that align with the observed transcriptional regulatory networks.

**FIGURE 2:**
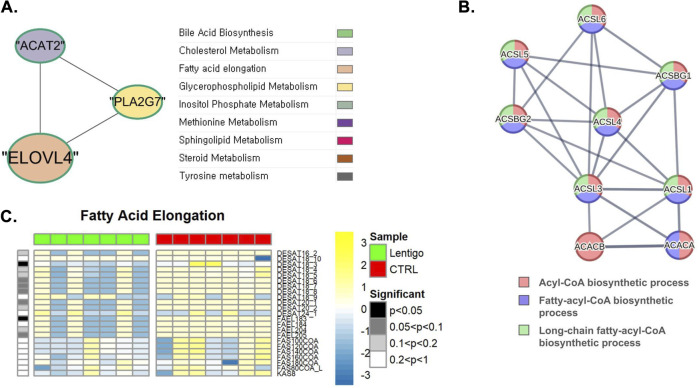
Integrated multi-omics analysis of fatty acid elongation in solar lentigo (SL). **(A)** Gene network analysis highlighting connections between ELOVL4, PLA2G7, and ACAT2, showing coordinated regulation between fatty acid elongation, phospholipid metabolism, and cholesterol synthesis. **(B)** PPI network demonstrating downregulation of ACACA, ACACB, ACSBG1, and ACSL5, which form a module involved in acyl-CoA biosynthesis, suggesting impaired fatty acid activation. **(C)** Metabolic flux simulation reveals decreased FAEL183 and DESAT18_3 reaction activity, indicating impaired elongation and desaturation of long-chain fatty acids, which may compromise membrane fluidity and energy balance. Statistical significance determined by Van der Waerden test (FDR-adjusted p < 0.05). Analysis based on RNA-seq data from SL lesions and control skin (n = 7 per group).

**FIGURE 3:**
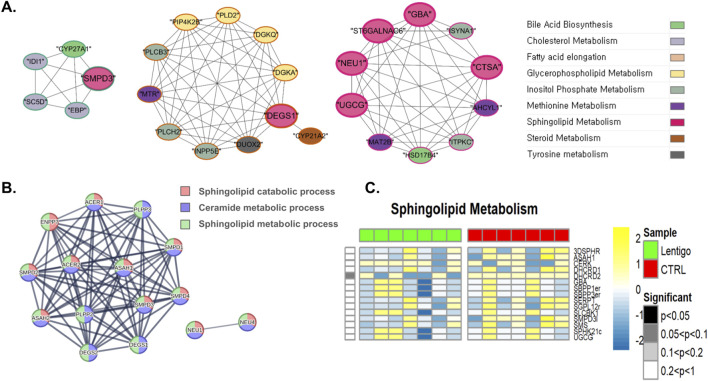
Integrated multi-omics analysis of sphingolipid metabolism in solar lentigo (SL). **(A)** Co-expression network of sphingolipid metabolic genes including SMPD3, DEGS1, and UGCG, highlighting crosstalk with glycerophospholipid and methionine metabolism. **(B)** PPI network showing upregulation of DEGS1 and DEGS2, reflecting compensatory mechanisms counteracting reduced DHCRD2 reaction activity. **(C)** Metabolic flux analysis showing significant reduction in DHCRD2 reaction activity, indicating diminished ceramide biosynthesis and altered redox-dependent sphingolipid regulation, potentially impairing skin barrier function. Statistical significance determined by Van der Waerden test (FDR-adjusted p < 0.05). Analysis based on RNA-seq data from SL lesions and control skin (n = 7 per group).

As shown in Figure 2C, our metabolic flux simulation of fatty acid metabolism in the SL revealed a substantial downregulation of the stearoyl-CoA desaturase reaction (BiGG ID: DESAT18_3), suggesting impaired fatty acid desaturation and elongation processes that may alter membrane rigidity. This reaction converts stearoyl-CoA to oleoyl-CoA. Stearoyl-CoA desaturase is critical for fatty acid desaturation, an essential process for maintaining lipid composition and membrane fluidity (Ntambi, 1999). Another reaction, dihydroceramide desaturase reaction (BiGG ID: DHCRD2), of the sphingolipid pathway showed a predominant reduction in SL, where the reduction in the activity of dihydroceramide desaturase led to a decline in ceramide production (Figure 3C). Dihydroceramide desaturase plays a pivotal role in the enzymatic conversion of dihydroceramide to ceramide, which is crucial for maintaining the equilibrium between sphingolipids and dihydrosphingolipids. In contrast, the upregulation identified through PPI analyses can be attributed to the increased transcription and translation of dihydroceramide desaturase, which serves as a compensatory mechanism for diminished DHCRD2 reaction. As shown in Figure 3B (*see also* Supplementary Figure S2), our PPI analysis revealed the upregulation of DEGS1 and DEGS2, both annotated as EC:1.14.19.17 and EC:1.14.18.5, respectively, in the KEGG database. These enzymes likely function in compensatory mechanisms to mitigate the reduced activity of dihydroceramide desaturase, thereby preserving cellular homeostasis. Such compensatory responses are frequently observed in patients with dermatological disorders (Coelho et al., 2015).

The complex interplay within the lipid metabolism was further illustrated by gene network analyses, as shown in Figure 2A (fatty acids) and Figure 3A (sphingolipids). Figure 2A shows that ELOVL4, crucial for the synthesis of very long-chain fatty acids (VLCFAs) necessary for ceramide production, is connected to *PLA2G7*, a gene related to glycerophospholipid metabolism, and *ACAT2*, a gene related to cholesterol metabolism. Figure 3A depicts a broader network, particularly within sphingolipid metabolism, which includes interactions involving *SMPD3* (involved in ceramide generation), *DEGS1* (dihydroceramide desaturase, critical for synthesizing ceramide from dihydroceramide), and *UGCG* (which utilizes ceramide), along with their connections to genes involved in other lipid pathways. These network analysis results collectively highlight the integrated regulation of pathways essential for maintaining sphingolipid homeostasis and membrane integrity in SL.

### 3.2 Disruption of oxidative stress management

**FIGURE 4:**
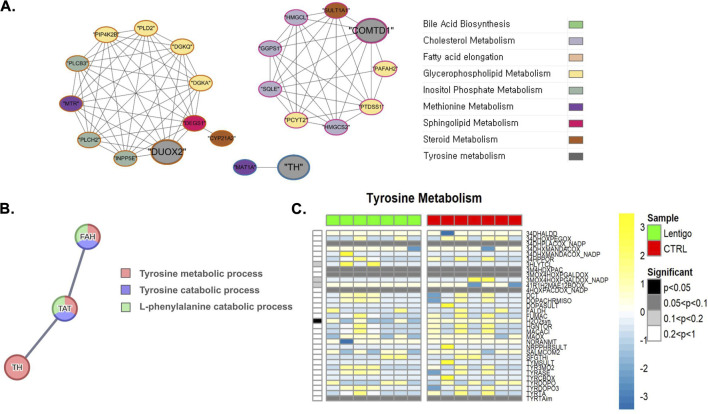
Integrated multi-omics analysis of tyrosine metabolism in solar lentigo (SL). **(A)** Gene co-expression network identifying interactions between DUOX2, COMTD, and TH, emphasizing redox regulation of tyrosine metabolism. **(B)** PPI network confirming upregulation of tyrosine hydroxylase (TH), the rate-limiting enzyme in L-DOPA biosynthesis, which drives melanogenesis. **(C)** Flux simulation reveals downregulation of the H2O2syn reaction, indicating altered hydrogen peroxide production and disrupted redox balance during melanogenic activation. Statistical significance determined by Van der Waerden test (FDR-adjusted p < 0.05). Analysis based on RNA-seq data from SL lesions and control skin (n = 7 per group).

Having recognized that oxidative stress disrupts the balance of the cellular redox state, affecting homeostasis and cellular responses in the SL, our analysis of sphingolipid metabolism revealed a predominant downregulation of the DHCRD2 reaction in the SL (Figure 3C). This reaction relies on FADH2 as a cofactor, and its enzymatic function is influenced by the presence of O_2_ and NAD(P)H. These observations highlight the bidirectional interactions between the cellular redox environment and dihydroceramide desaturase activity (Fabrias et al., 2012). Similarly, in tyrosine metabolism, SL samples displayed a substantial downregulation of the hydrogen peroxide synthesis reaction (BiGG ID: H2O2syn), where the reaction is facilitated by the enzyme hydrogen peroxide synthase, which is dependent on NADPH, and converts oxygen (O_2_), NADPH, and protons (H^+^) into hydrogen peroxide (H_2_O_2_) and NADP^+^(Figure 4C). Hydrogen peroxide is essential for modulating the cellular redox state and functions as a signaling molecule that is critical for preserving homeostasis and cellular responses. Further insights into these metabolic shifts were provided by network analysis (Figures 3A, 4A). The sphingolipid network analysis (Figure 3A), consistent with the alterations in dihydroceramide desaturase activity, reaffirmed the complex regulatory interactions among the key enzymes governing ceramide synthesis and turnover. More pertinent to the changes in tyrosine metabolism, Figure 4A depicts interactions involving *DUOX2* (relevant to H_2_O_2_ balance) and *COMTD* (linked to L-DOPA metabolism), and importantly, visualized tyrosine hydroxylase (*TH*), the pivotal enzyme for L-DOPA production from tyrosine. PPI analysis further supported this upregulation by revealing increased translation of the TH gene (EC:1.14.16.2), highlighting its enhanced role in tyrosine metabolism, as shown in Figure 4B and Supplementary Figure S3. Increased tyrosine hydroxylase activity promoted melanogenesis, resulting in increased the production of reactive oxygen species (ROS) (Iyengar and Misra, 1987). These observations suggest that tyrosine metabolism plays a significant role in the management of oxidative stress in SL.

### 3.3 PTEN-mediated PI3K/Atk pathway disruption and calcium homeostasis alterations

**FIGURE 5:**
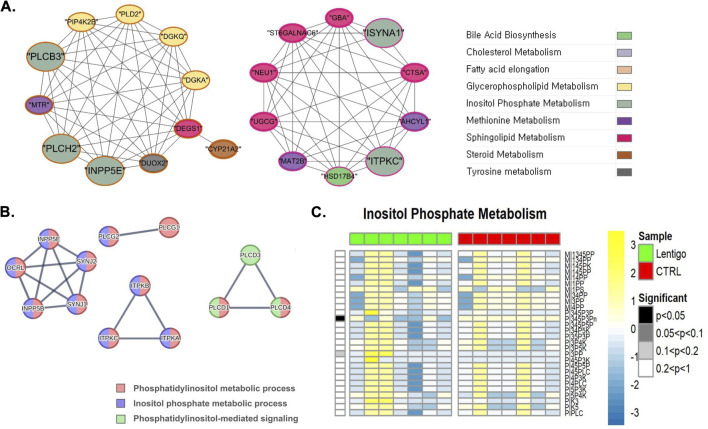
Integrated multi-omics analysis of inositol phosphate metabolism in solar lentigo (SL). **(A)** Gene co-expression network highlighting INPP5E, PLCB3, and ITPKC as regulatory hubs linking inositol phosphate turnover with PI3K/Akt and calcium signaling. **(B)** PPI network emphasizing phospholipase C (PLC) isoforms as key mediators of phosphatidylinositol metabolism. **(C)** Metabolic flux simulation showing decreased PI(3,4,5)P3 phosphatase reaction (PI345P3Pn) activity driven by PTEN downregulation, indicating impaired PI3K/Akt signaling and dysregulated calcium homeostasis. Statistical significance determined by Van der Waerden test (FDR-adjusted p < 0.05). Analysis based on RNA-seq data from SL lesions and control skin (n = 7 per group).

As illustrated in Figure 5C, our metabolic simulation analysis exhibited a considerable reduction in the PI(3,4,5)P_3_ phosphatase reaction (BiGG ID: PI345P3Pn) in SL, where the reaction is facilitated by phosphatidylinositol-3,4,5-trisphosphate 3-phosphatase, an enzyme encoded by the *PTEN* gene. *PTEN* is integral to the regulation of a variety of cellular processes, including cell metabolism, by modulating Akt activity via the PTEN/PI3K/Akt signaling pathway (Downward, 2004). Further insights at the network level were provided by gene coexpression analysis (Figure 5A). This analysis highlights the key enzymes within inositol phosphate metabolism (greyish-green nodes) and their interactions. Notably, *INPP5E*, an inositol polyphosphate-5-phosphatase involved in modulating PI3K/Akt signaling by regulating PIP levels, is part of a large cluster interconnected with glycerophospholipid (e.g., *PLCB3*) and sphingolipid metabolism genes. The network also prominently featured enzymes critical for calcium signaling. Phospholipase C isoforms, such as *PLCB3* (a key generator of IP_3_ and diacylglycerol (DAG) from PIP_2_) and *PLCH2,* formed a module. In contrast, *ITPKC* (inositol-trisphosphate 3-kinase), which influences IP_3_ levels and calcium signals, was observed in a separate cluster interacting with sphingolipid and methionine metabolism genes. These visualized networks underscore the complex interplay and co-regulation of proteins involved in both PI3K/Akt pathway modulation and calcium signaling through inositol phosphates.

### 3.4 Impaired fatty acid elongation and energy metabolism in solar lentigo

In SL samples, membrane fluidity maintenance and fatty acid elongation were predominantly inhibited. In the context of fatty acid metabolism, pronounced reduction of the fatty-acyl-CoA elongation reaction (BiGG ID: FAEL183) was observed, indicating a decrease in the elongation activity of fatty acids (Figure 2C). This reaction, catalyzed by a fatty-acyl-CoA elongation enzyme, converts linoleoyl-CoA to dihomo-γ-linolenoyl-CoA using malonyl-CoA as a substrate.

Additionally, *ACACA* and *ACACB* (EC:6.4.1.2), which convert acetyl-CoA to malonyl-CoA, showed suppressed expression in SL (Supplementary Figure S4). Malonyl-CoA not only acts as a key substrate for fatty acid elongation but also serves as a regulatory molecule for fatty acid oxidation (Bowman and Wolfgang, 2019). Therefore, a decreased activity of these enzymes is likely to impair both processes.

PPI analysis, as shown in Figure 2B, supported this observation by revealing that ACACA, ACACB, and multiple long-chain acyl-CoA synthetases, such as ACSBG1 and ACSL5, form a closely connected module within the acyl-CoA biosynthetic process. These enzymes, many of which were downregulated, are central to fatty acid activation, further supporting the functional suppression of SL.

**FIGURE 6:**
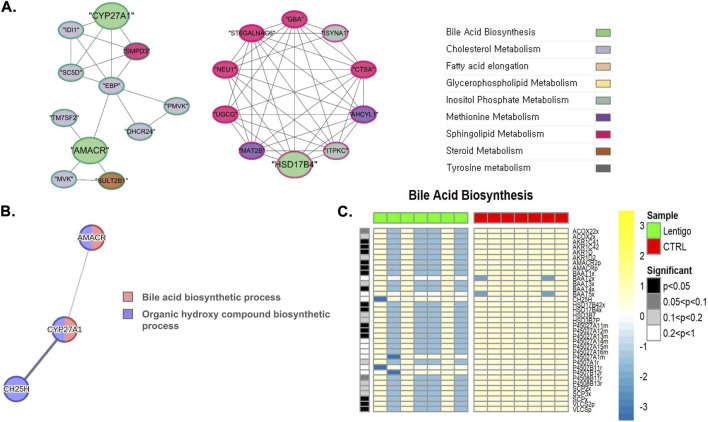
Integrated multi-omics analysis of bile acid biosynthesis in solar lentigo (SL). **(A)** Gene network showing CYP27A1, AMACR, and HSD17B4 interaction with cholesterol and sphingolipid metabolic genes, revealing pathway interconnectivity. **(B)** PPI network and flux analysis demonstrating coordinated downregulation of CH25H, CYP27A1, and AMACR, implying multi-step suppression of bile acid biosynthesis and reduced cholesterol turnover. **(C)** Metabolic flux simulation revealing reduced oxidation and hydroxylation fluxes mediated by CYP27A1 and AMACR, demonstrating impaired bile acid production and disrupted cholesterol turnover in SL. Statistical significance determined by Van der Waerden test (FDR-adjusted p < 0.05). Analysis based on RNA-seq data from SL lesions and control skin (n = 7 per group).

Similarly, disruption of bile acid metabolism was observed. Figure 6A shows that CYP27A1, AMACR, and HSD17B4, which are essential enzymes in bile acid biosynthesis, exhibited notable interactions with genes involved in cholesterol and sphingolipid metabolism. This network suggests that bile acid metabolism does not operate in isolation but rather interfaces with broader lipid regulatory pathways.

Additionally, Figure 6B and Supplementary Figure S5 show the coordinated suppression of CH25H (EC:1.14.99.38), CYP27A1 (EC:1.14.15.15), and AMACR (EC:5.1.99.4), which act sequentially during bile acid biosynthesis. This pattern implies multi-step disruption of the pathway and potential downstream effects on cholesterol turnover.

Collectively, these findings indicate that SL is characterized by broad dysregulation of fatty acid metabolism, encompassing elongation, activation, and oxidation, likely leading to altered lipid composition and signaling.

### 3.5 Cholesterol biosynthesis modification and sterol homeostasis dysfunction

**FIGURE 7:**
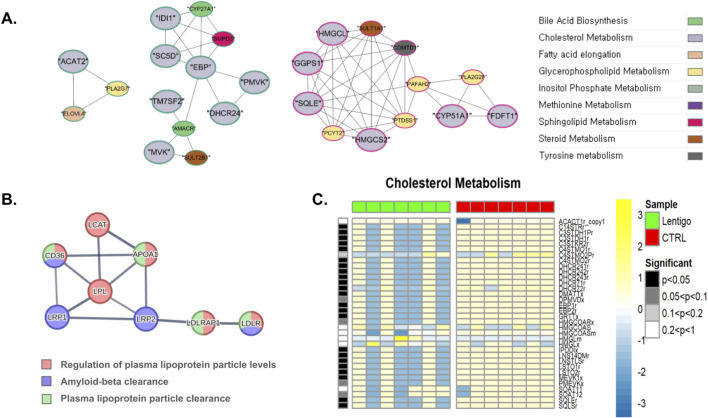
Integrated multi-omics analysis of cholesterol metabolism in solar lentigo (SL). **(A)** Gene network analysis illustrating clusters of cholesterol biosynthetic enzymes (DHCR7, DHCR24, MVK, PMVK, CYP51A1), indicating coordinated transcriptional suppression. **(B)** PPI network highlighting downregulation of cholesterol transport proteins LDLR, LRP1/2, and APOA1, reflecting impaired lipoprotein trafficking. **(C)** Metabolic flux simulation showing decreased activity of SQLSr, SQLEr, and DHCR7/24 reactions, suggesting inhibition of cholesterol biosynthesis and compromised skin barrier maintenance. Statistical significance determined by Van der Waerden test (FDR-adjusted p < 0.05). Analysis based on RNA-seq data from SL lesions and control skin (n = 7 per group).

Figure 7C shows a substantial downregulation of key cholesterol biosynthesis reactions in SL. Specifically, the squalene synthase reaction (BiGG ID: SQLSr) and squalene epoxidase endoplasmic reticular NADP reaction (BiGG ID: SQLEr) were diminished, indicating a decreased conversion of farnesyl pyrophosphate to squalene, and subsequently, squalene to 2,3-oxidosqualene. Squalene epoxidase (SQLE) is responsible for the stereospecific conversion of squalene to 2,3(S)-oxidosqualene, which is the initial oxygenation step in cholesterol biosynthesis. Its downregulation may hinder cholesterol synthesis and disturb cholesterol homeostasis (Padyana et al., 2019). In addition, DHCR71r reaction, DHCR72r reaction, DHCR241r reaction, DHCR242r reaction, and DHCR243r reaction, which are catalyzed by 7-dehydrocholesterol reductase and 24-dehydrocholesterol reductase, were reduced. These reactions are pivotal in the terminal phases of cholesterol biosynthesis and facilitate the conversion of 7-dehydrocholesterol and desmosterol to cholesterol. As shown in Figure 7A, gene network analysis further underscored the coordinated nature of cholesterol metabolism. This analysis revealed distinct clusters of functionally associated genes central to cholesterol biosynthesis (nodes predominantly colored light gray), including key enzymes, such as *TM7SF2* (DHCR7), *DHCR24*, *MVK*, *PMVK*, and *CYP51A1*. These clusters demonstrate their interconnectedness within the cholesterol synthesis pathway and their links to other lipid metabolic processes, such as sphingolipid and bile acid biosynthesis.

Translation-level analysis indicated a considerable reduction in the levels of critical proteins involved in cholesterol transport, as shown in Supplementary Figure S6. Complementing these findings, PPI analysis was performed to elucidate the interaction networks of these key cholesterol transport-related proteins (Figure 7B). This network highlighted *LDLR*, *LRP1*, and *LRP2* receptors crucial for LDL uptake and their association with amyloid-beta and plasma lipoprotein particle clearance (nodes colored blue and/or green, reflecting their pathway involvement). The same PPI network underscored the centrality of *APOA1*, the main high-density lipoprotein (HDL), in the regulation of plasma lipoprotein levels and ‘Plasma lipoprotein particle clearance (red and/or green nodes). The observed downregulation of these interconnected proteins, LDLR and LRPs for LDL uptake, and APOA1 for HDL formation and reverse cholesterol transport, significantly impaired cholesterol homeostasis. For instance, diminished APOA1 impairs HDL formation, reduces cholesterol efflux from peripheral tissues to the liver, leads to lipid dysregulation, and diminishes the ability of HDL to modulate immune cell function, potentially worsening inflammatory and autoimmune conditions (Trakaki and Marsche, 2020).

## 4 Discussion

Lipid metabolism and the cellular redox state are intimately linked by multiple biochemical processes that are relevant to the pathophysiology of SL. The changes in sphingolipid and tyrosine metabolism described above imply a complex interplay between lipid dysregulation and redox modulation of oxidative stress in diseased UV skin.

Reduction of the response of DHCRD2 reactions to sphingolipid metabolism results in loss of redox balance in SL lesions. Dihydroceramide desaturase activity is dependent on FADH2 cofactor, O_2_ availability, and NAD(P)H, setting up a dynamic interaction between cellular redox state and sphingolipid homeostasis (Fabrias et al., 2012; Łuczaj et al., 2020). Sphingolipid metabolism is critical for the resistance of cells to oxidative stress because sphingolipid-preserving enzymes, such as CERKL, have been shown to protect skin cells from oxidative damage and maintain ceramide synthesis under stress conditions (Meyer et al., 2021; Dini et al., 2021). The inhibition of DHCRD2 reaction may compromise the antioxidant protective mechanisms of the skin, potentially increasing UV-induced cell damage.

Network analysis of the interactions between COMTD1, DUOX2, and TH revealed complex redox regulation in SL. DUOX2, which maintains the balance of hydrogen peroxide, appears to be coordinately regulated by the induction of TH (Kumar et al., 2018). TH activity activated by enhancement of PPI analysis for enhanced gene translation promotes L-DOPA formation from tyrosine required for melanogenesis, but simultaneously produces reactive oxygen species (ROS) (Li et al., 2024; Chen J. et al., 2024). This creates a paradox in the sense that higher production of pigments creates more oxidative stress because melanogenesis is a process that generates ROS as a byproduct of tyrosinase action (Kim et al., 2024).

Despite the apparent decrease of H_2_O_2_ production via the H2O2syn reaction, the upregulation of TH signals a shift towards increased melanogenic activity capable of surpassing cellular antioxidant defenses. Downregulation of tyrosine aminotransferase and fumarylacetoacetase conjugately manifests as decreased tyrosine catabolism, potentially leading to tyrosine accumulation and further worsening oxidative stress-associated damage in melanocytes (Conner, 2021; Luo et al., 2023a). This metabolic derangement may contribute to the characteristic hyperpigmentation and cellular dysfunction of solar lentigines.

The interconnectedness of these metabolic alterations emphasizes the bidirectional association between lipid homeostasis and oxidative stress management in SL pathogenesis (Vecchio et al., 2021; Jenkins et al., 2013; Speeckaert et al., 2023). The concurrent disruption of sphingolipid-protective mechanisms and tyrosine metabolic homeostasis suggests that SL provides dysfunctional cellular protection against UV-induced oxidative injury.

Coordinated alterations in membrane lipid composition observed in SL reflect a fundamental shift in cellular adaptation to chronic UV exposure. The enhanced conversion of phosphatidylserine to phosphatidylcholine suggests an adaptive response to maintain membrane stability under oxidative stress, because PC is more resistant to lipid peroxidation than PE (Zhu et al., 2024). This methylation-dependent pathway may represent a protective mechanism against UV-induced membrane damage, particularly in photodamaged skin where oxidative stress is persistent (Patra et al., 2023).

Gene co-expression network analysis revealed a sophisticated regulatory architecture underlying membrane remodeling in SL. Key glycerophospholipid enzymes form distinct functional clusters with DGKQ, demonstrating strong co-expression with PLD2, whereas PTDSS1 shows coordinated regulation with PAFAH2 (Wu and Zhao, 2024). These specific co-expression patterns indicate that membrane phospholipid synthesis is governed by tightly regulated gene modules that respond in coordination with UV-induced stress. The extensive crosstalk between glycerophospholipid metabolism and sphingolipid pathways, particularly through DEGS1 connections, demonstrates the integrated nature of membrane lipid homeostasis in photodamaged skin (Liu et al., 2023).

The reduction of DHCRD2 reaction in sphingolipid metabolism compromises ceramide synthesis, potentially undermining the lipid barrier function of the skin. The compensatory upregulation of DEGS1 and DEGS2 indicates that SL lesions attempt to preserve sphingolipid homeostasis, despite metabolic dysfunction (Tessema et al., 2018). However, this compensation appears insufficient, as ceramide deficiency remains a characteristic feature of aged and photodamaged skin. The inability to fully restore ceramide levels may explain the compromised barrier function and increased susceptibility to environmental stressors observed in solar lentigines (Ma et al., 2020).

Overexpression of PLA2 family enzymes indicates active phospholipid turnover and membrane remodeling in SL. While potentially contributing to inflammatory signaling, this increased PLA2 activity may also represent an attempt to remodel damaged membrane components and facilitate cellular repair processes (Dalmau et al., 2018). The central role of PLA2G7 as a regulatory hub connecting glycerophospholipid, cholesterol, and fatty acid metabolism suggests that SL represents a systemic reorganization of membrane metabolism rather than isolated pathway disruption.

Impairment of cholesterol biosynthesis through reduced SQLE, DHCR7, and DHCR24 activity disrupts this essential barrier component. Cholesterol plays a crucial role in skin barrier formation and keratinocyte differentiation and its synthesis is tightly regulated by sterol regulatory element-binding proteins (Wang et al., 2020). Coordinated downregulation of cholesterol transport proteins LDLR, LRP1, LRP2, and APOA1 further exacerbates cholesterol deficiency in SL lesions, compromising membrane structure and barrier integrity (Muresan et al., 2019). This cholesterol dysregulation may contribute to altered membrane fluidity and compromised intercellular communication between keratinocytes and melanocytes, potentially influencing the characteristic hyperpigmentation pattern of solar lentigines.

The overall dysregulation of fatty acid metabolism in SL represents a fundamental alteration in cellular energy homeostasis, which is more than simply pigmentation. The drastic reduction of the FAEL183 reaction defines impairment in conversion of linoleoyl-CoA to dihomo-γ-linolenoyl-CoA, disrupting very-long-chain fatty acid synthesis required for cellular energy production and membrane integrity (Oh et al., 2022).

The interaction network between ELOVL4, PLA2G7, and ACAT2 demonstrates the coordinated regulation of fatty acid elongation with phospholipid and cholesterol metabolism. The cross-talk suggests that SL involves the synchronized disruption of multiple lipid biosynthetic pathways essential for maintaining cellular energy homeostasis (Luo et al., 2020).

The suppressed expression of ACACA and ACACB is the primary bottleneck in cellular energy metabolism. Both enzymes catalyze acetyl-CoA to malonyl-CoA, which is not only the immediate substrate for the elongation of fatty acids but also a primary regulatory molecule suppressing the oxidation of fatty acids (Tu et al., 2024). The dual role of malonyl-CoA is that reduced ACACA/ACACB activity has a cascade effect, both suppressing fatty acid synthesis and disrupting the balance between lipid anabolism and catabolism, with the ultimate effect of sabotaging cellular energy production (Papaccio et al., 2024).

Protein-protein interaction analysis showed that a tightly connected module of ACACA, ACACB, and various long-chain acyl-CoA synthetases (ACSBG1 and ACSL5) underscores the concertedness of fatty acid activation defects in SL. All these enzymes play critical roles in conjugating fatty acids to energy-rich acyl-CoA derivatives for cellular metabolism. Coordinated downregulation of this enzyme module shows that SL lesions experience a systematic disruption of fatty acid utilization, potentially leading to energy deficiency and cellular dysfunction (Yu et al., 2024).

In the pathogenesis of SL, changes in fatty acid metabolism can exert profound effects on energy homeostasis and pigment production in melanocytes. Fatty acid metabolism provides the energy substrates required for melanogenesis and melanosome transport (Chen X. et al., 2024). The disruption noted in fatty acid synthesis and activation can therefore contribute to the metabolic dysregulation that characterizes solar lentigines (Li et al., 2025).

Extreme repression of bile acid synthesis, as indicated by the downregulation of CYP27A1 and AMACR, is another facet of metabolic failure in SL. Their repression in a coordinated manner indicates multi-step disruption that can lead to cholesterol accumulation and deranged energy metabolism. Such coordinated disruption of both fatty acid and bile acid metabolism is reflective of a fundamental change in energy homeostasis that may be implicated in UV-induced skin aging pathophysiology.

Simulation analysis of metabolic flux showed multifaceted dysregulation of the PI3K/Akt pathway in SL, where PTEN, an important tumor suppressor that is commonly mutated in most skin cancers, such as melanoma (Sun et al., 2024; Chen et al., 2021), was mostly downregulated. PTEN functions as a suppressive modulator of the PI3K/Akt signaling pathway by dephosphorylation of PIP_3_ to PIP_2_, and its suppression results in the constitutive activation of downstream oncogenic pathways that have been implicated in various dermatological conditions like acne, psoriasis, and skin cancers (Lee et al., 2021; Namiki et al., 2015).

Gene coexpression network analysis identified INPP5E, an inositol polyphosphate-5-phosphatase, as a regulatory hub node in a large interconnected cluster. INPP5E controls PI3K/Akt signaling by modulating phosphoinositide levels and network integration with glycerophospholipid metabolism genes, suggesting coordinated regulation of lipid signaling pathways in SL pathogenesis (Scortegagna et al., 2015). The upregulated INPP5E activity in SL is a compensatory mechanism that phosphohydrates PIP_3_ into PIP_2_, thereby conferring tumor-suppressing activities as observed in melanoma, where enzymatic activity is used to curtail malignant transformation (Conde-Perez et al., 2015) (Supplementary Figure S7).

Network analysis also focused on the central role of the PLC isoforms PLCB3 and PLCH2, which are unique functional modules in the co-expression network. The activation of PLC consumes PIP_2_ to generate inositol 1,4,5-trisphosphate (IP_3_) and DAG, which may reduce the level of PIP_3_ and indirectly suppress PI3K/Akt signaling. This double mechanism—direct dephosphorylation of PIP_3_ by phosphatases and indirect reduction through PLC consumption of PIP_2_—defines a coordinated inhibition of pro-proliferative signaling in SL lesions.

Calcium signaling is another major parallel pathway affected in SL, with deep alterations in intracellular calcium homeostasis-regulating enzymes. Activation of the observed PLC and network positioning of ITPKC (inositol-trisphosphate 3-kinase) within a separate cluster that is in contact with sphingolipid and methionine metabolism genes indicates a very complex regulatory network controlling IP_3_ concentration and subsequent intracellular calcium release. In melanocytes, PLC plays a central role in UV light detection and cellular response signaling pathways (Wang et al., 2021), whereas calcium signaling is essential for UV-stimulated melanogenesis and subsequent melanin transfer to keratinocytes (Luo et al., 2023b).

The disruption of calcium homeostasis in the SL is of particular significance owing to its double biological function. In healthy individuals, controlled calcium signaling enhances melanogenesis via tyrosinase activation and melanosome transport (Luo et al., 2023b). However, in the context of chronic UV exposure, a characteristic of SL development, hypercalcemic influx could trigger oxidative stress mechanisms and participate in the pathological pigmentation patterns of such lesions (Kim and Lee, 2023). The network-based identification of ITPKC within a metabolically distinct cluster suggests a putative regulatory function in calcium signaling, which may be impaired in SL, possibly leading to dysregulated IP_3_ turnover and altered calcium dynamics.

The intersection of these findings indicates that SL pathogenesis represents a synergistic dysregulation of both the PI3K/Akt and calcium signaling pathways by virtue of the complex interactions between phosphoinositide metabolism, PLC activation, and inositol phosphate regulatory enzymes.

This comprehensive discussion of multi-systemic dysregulation reveals that SL pathogenesis is an orchestrated dysregulation of lipid metabolism beyond melanin overproduction, including orchestrated dysregulation of fatty acid elongation, sphingolipid homeostasis, and membrane structure. Downregulation of key enzymes, such as ACACA/ACACB, fatty-acyl-CoA elongation enzyme and dihydroceramide desaturase, and defective PI3K/Akt and calcium signaling pathways indicate drastic changes in cellular energy metabolism and redox regulation following chronic UV exposure. These metabolic alterations suggest that lipid pathway disruption is a may represent an important mechanism in UV-induced photoaging and reveal novel targets for the treatment of pigmentary diseases. Identification of metabolic chokepoints for fatty acid and sphingolipid production could guide the design of lipid-targeted therapies to prevent and treat SL.

Notwithstanding these significant findings regarding lipid metabolic dysregulation in SL, several inherent study constraints require careful consideration. The relatively small sample size (n = 7 pairs) may limit the generalizability of these results to broader populations with SL. Additionally, our conclusions are primarily derived from transcriptomic data and computational modeling approaches. Direct experimental validation though targeted metabolic profiling or functional biochemical assays would substantially strengthen the mechanistic interpretations presented. Nevertheless,the consistent patterns observed across multiple metabolic pathways suggest robust detection of major pathway alterations. Future research employing larger cohorts with experimental validation will be essential to validate these findings and to examine lipid-targeted therapeutic strategies in clinical dermatology.

## Data Availability

The datasets presented in this study can be found in online repositories. The names of the repository/repositories and accession number(s) can be found below: NCBI Gene Expression Omnibus (GEO), GSE318414 https://www.ncbi.nlm.nih.gov/geo/query/acc.cgi?acc=GSE318414.

## Supplementary material

SUPPLEMENTARY FIGURE S1 Glycerophospholipid metabolism pathway map. KEGG pathway diagram showing comprehensive glycerophospholipid metabolic network with metabolic flux changes in solar lentigo. Color-coded reactions indicate upregulation (red) and downregulation (green) based on constraint-based flux simulation. Key enzymes and intermediates are labeled with KEGG identifiers. This detailed pathway view supports the glycerophospholipid findings presented in Figure 1.

SUPPLEMENTARY FIGURE S2 Sphingolipid metabolism pathway map. KEGG pathway diagram illustrating complete sphingolipid biosynthetic and catabolic pathways with metabolic flux alterations in solar lentigo lesions. Color scale represents log2 fold changes in reaction activities, highlighting reduced ceramide synthesis (DHCRD2) and compensatory mechanisms. Pathway includes ceramide, sphingomyelin, and glycosphingolipid branches supporting Figure 3 analysis.

SUPPLEMENTARY FIGURE S3 Tyrosine metabolism pathway map. Comprehensive KEGG pathway showing tyrosine catabolism and melanin biosynthesis with metabolic flux changes in solar lentigo. Enhanced tyrosine hydroxylase activity and melanogenesis are evident, while tyrosine degradation pathways show suppression. Color-coded metabolites and reactions support the oxidative stress and melanogenic findings described in Figure 4.

SUPPLEMENTARY FIGURE S4 Fatty acid biosynthesis pathway map. KEGG pathway diagram displaying fatty acid synthesis and elongation networks with metabolic alterations in solar lentigo. Shows coordinated downregulation of acetyl-CoA carboxylase (ACACA/ACACB) and fatty acid elongation systems. Color-coded pathway components illustrate the systematic suppression of fatty acid metabolism supporting Figure 2 results.

SUPPLEMENTARY FIGURE S5 Primary bile acid biosynthesis pathway map. KEGG pathway showing cholesterol-to-bile acid conversion with metabolic flux changes in solar lentigo. Demonstrates coordinated suppression of key enzymes including CYP27A1, AMACR, and related hydroxylases. Color gradient indicates reduced bile acid production capacity, supporting the cholesterol turnover disruption described in Figure 6.

SUPPLEMENTARY FIGURE S6 Cholesterol metabolism pathway map. Comprehensive KEGG pathway diagram of cholesterol biosynthesis from acetyl-CoA with metabolic flux alterations in solar lentigo. Shows systematic downregulation of mevalonate pathway enzymes, squalene synthesis, and sterol ring formation. Color-coded reactions demonstrate the coordinated suppression of cholesterol biosynthesis presented in Figure 7.

SUPPLEMENTARY FIGURE S7 Inositol phosphate metabolism pathway map. KEGG pathway illustrating inositol phosphate signaling networks and phosphatidylinositol metabolism with flux changes in solar lentigo. Highlights PTEN-mediated PI3K/Akt pathway disruption and calcium signaling alterations. Color-coded metabolites show reduced PIP3 phosphatase activity and altered second messenger systems supporting Figure 5 findings.

## Glossary

ACACA: Acetyl-CoA carboxylase alpha
ACACB: Acetyl-CoA carboxylase beta
ACAT2: Acetyl-CoA acetyltransferase 2
ACSBG1: Acyl-CoA synthetase bubblegum family member 1
ACSL5: Acyl-CoA synthetase long-chain family member 5
AMACR: Alpha-methylacyl-CoA racemase
APOA1: Apolipoprotein A1
CH25H: Cholesterol 25-hydroxylase
COMTD1: Catechol-O-methyltransferase domain containing 1
CYP27A1: Cytochrome P450 family 27 subfamily A member 1
DAG: Diacylglycerol
DEGS1: Delta 4-desaturase, sphingolipid 1
DEGS2: Delta 4-desaturase, sphingolipid 2
DHCR7: 7-dehydrocholesterol reductase
DHCR24: 24-dehydrocholesterol reductase
DGKQ: Diacylglycerol kinase theta
DUOX2: Dual oxidase 2
EC: Enzyme Commission
ELOVL4: ELOVL fatty acid elongase 4
FDR: False discovery rate
GO: Gene Ontology
GSEA: Gene Set Enrichment Analysis
HDL: High-density lipoprotein
HSD17B4: Hydroxysteroid 17-beta dehydrogenase 4
INPP5E: Inositol polyphosphate-5-phosphatase E
**IP**_**3**_: Inositol 1,4,5-trisphosphate
IRB: Institutional Review Board
ITPKC: Inositol-trisphosphate 3-kinase C
KEGG: Kyoto Encyclopedia of Genes and Genomes
LDL: Low-density lipoprotein
LDLR: Low-density lipoprotein receptor
LRP1: LDL receptor related protein 1
LRP2: LDL receptor related protein 2
MVK: Mevalonate kinase
PAFAH2: Platelet activating factor acetylhydrolase 2
PC: Phosphatidylcholine
PE: Phosphatidylethanolamine
**PIP**_**2**_: Phosphatidylinositol 4,5-bisphosphate
**PIP**_**3**_: Phosphatidylinositol 3,4,5-trisphosphate
PLA2G7: Phospholipase A2 group VII
PLCB3: Phospholipase C beta 3
PLCH2: Phospholipase C eta 2
PMVK: Phosphomevalonate kinase
PPI: Protein-protein interaction
PS: Phosphatidylserine
PTEN: Phosphatase and tensin homolog
PTDSS1: Phosphatidylserine synthase 1
ROS: Reactive oxygen species
SL: Solar lentigo
SMPD3: Sphingomyelin phosphodiesterase 3
SQLE: Squalene epoxidase
STRING: Search Tool for the Retrieval of Interacting Genes/Proteins
TH: Tyrosine hydroxylase
TM7SF2: Transmembrane 7 superfamily member 2
TPM: Transcripts per million
UGCG: UDP-glucose ceramide glucosyltransferase
UV: Ultraviolet
