# Supplementary figures and images for: Lipid metabolism dysregulation in solar lentigo: a multi-system-level analysis reveals membrane instability and energy homeostasis disruption
Source: Front Cell Dev Biol. 2026 Mar 2;14:1751543. doi: 10.3389/fcell.2026.1751543 (PMC12989492; doi:10.3389/fcell.2026.1751543)

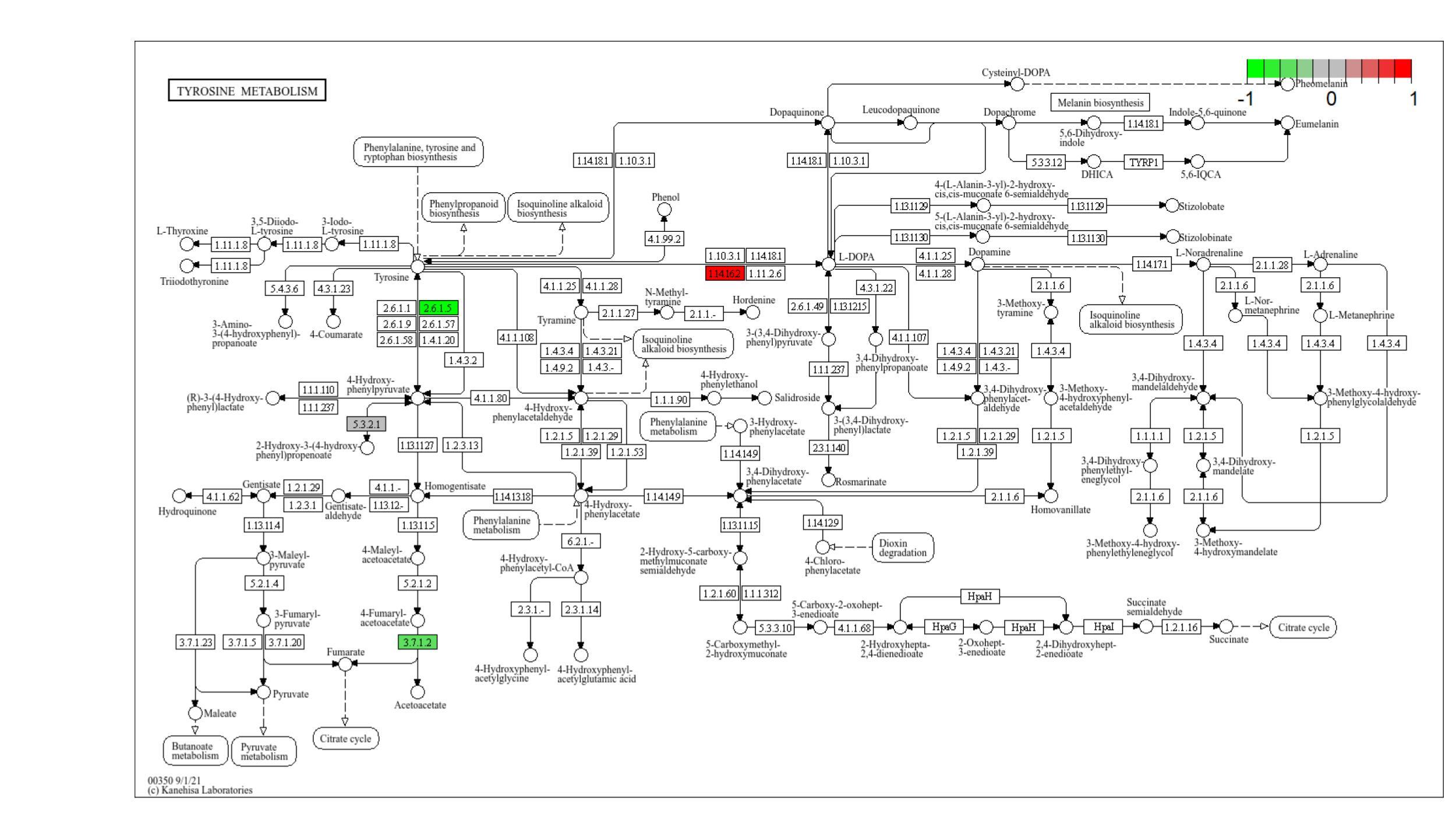

Supplement: Supplementary file 1 [file Image3.jpeg]

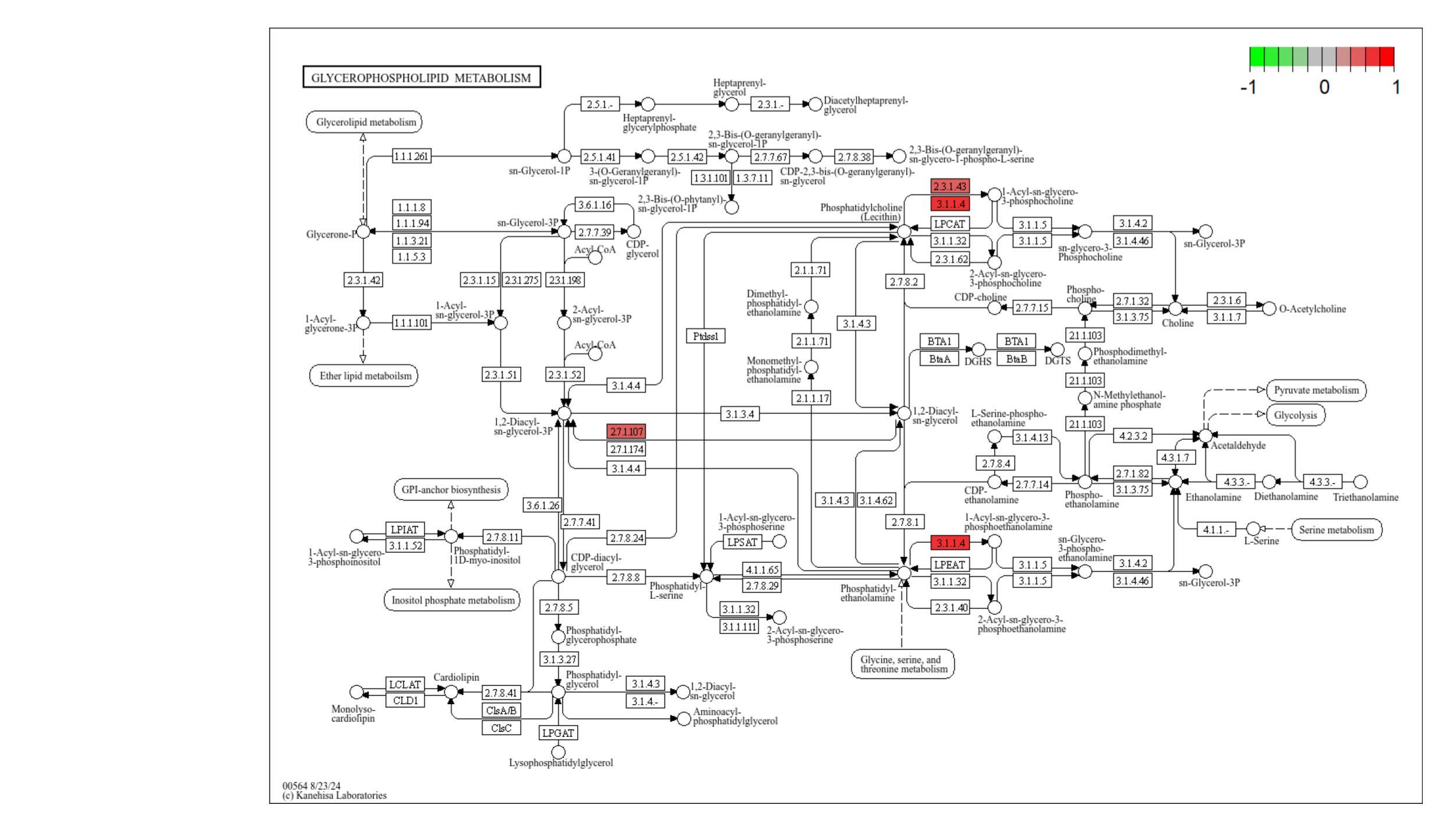

Supplement: Supplementary file 2 [file Image1.jpeg]

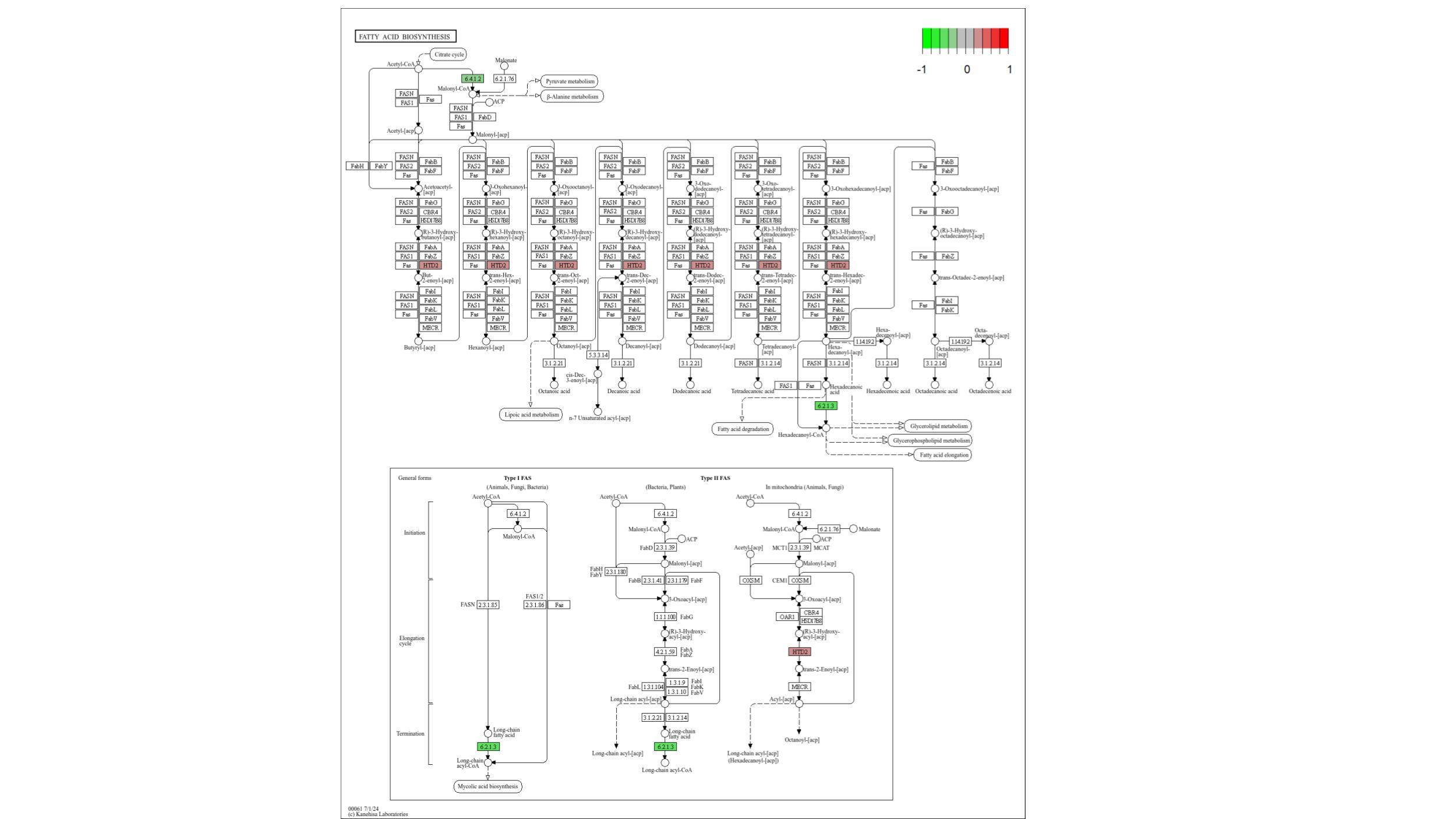

Supplement: Supplementary file 3 [file Image4.jpeg]

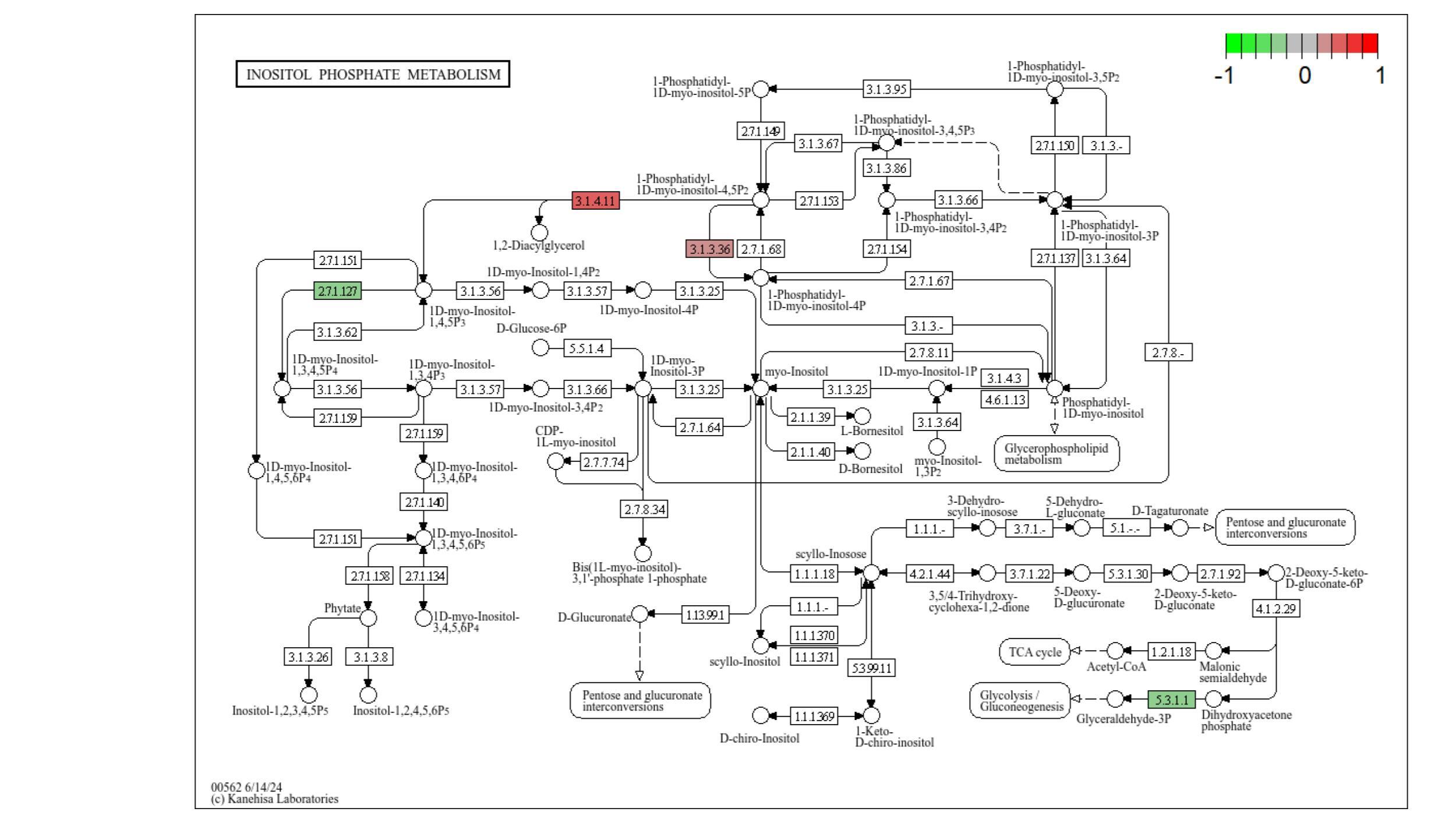

Supplement: Supplementary file 4 [file Image7.jpeg]

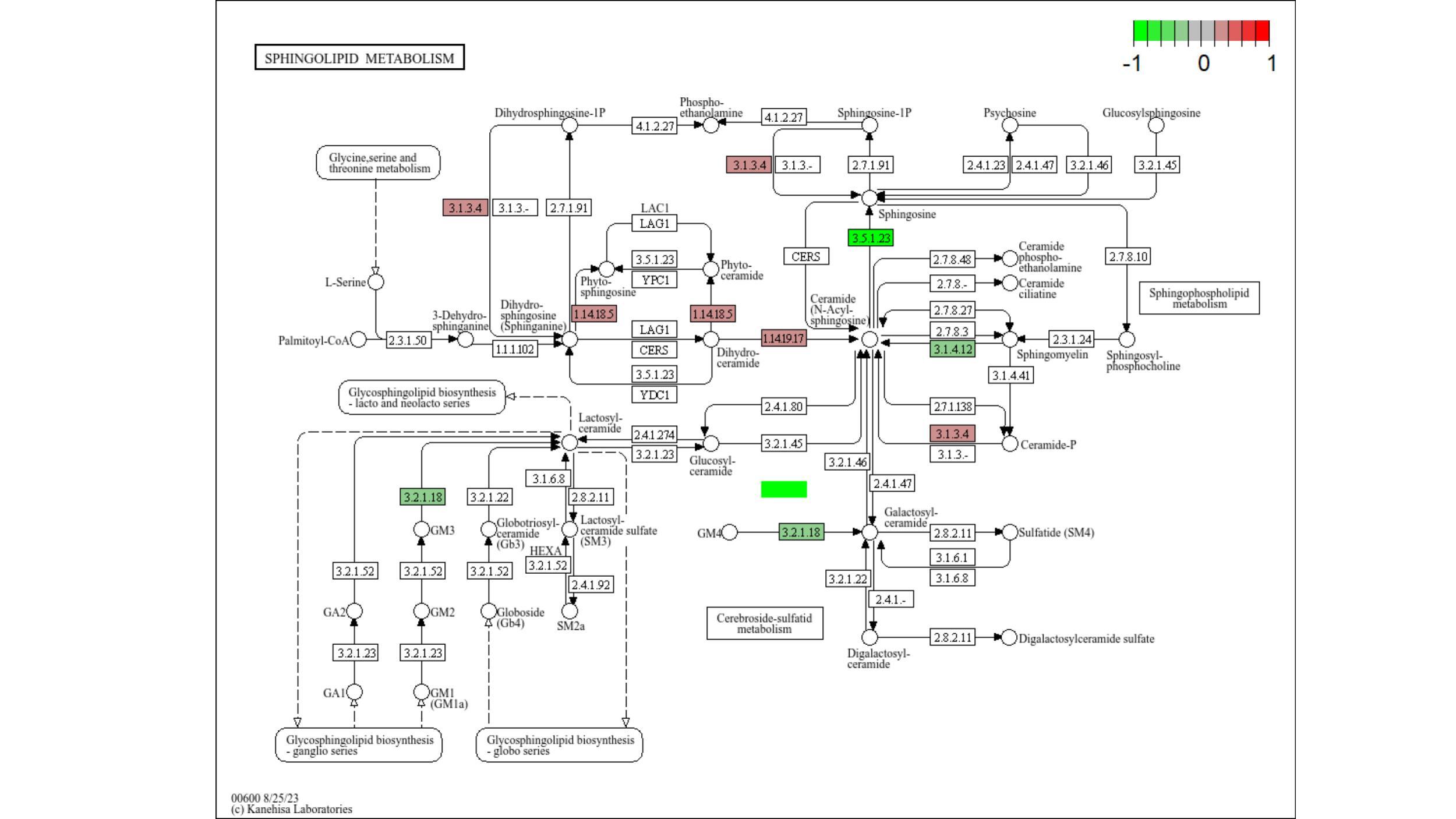

Supplement: Supplementary file 5 [file Image2.jpeg]

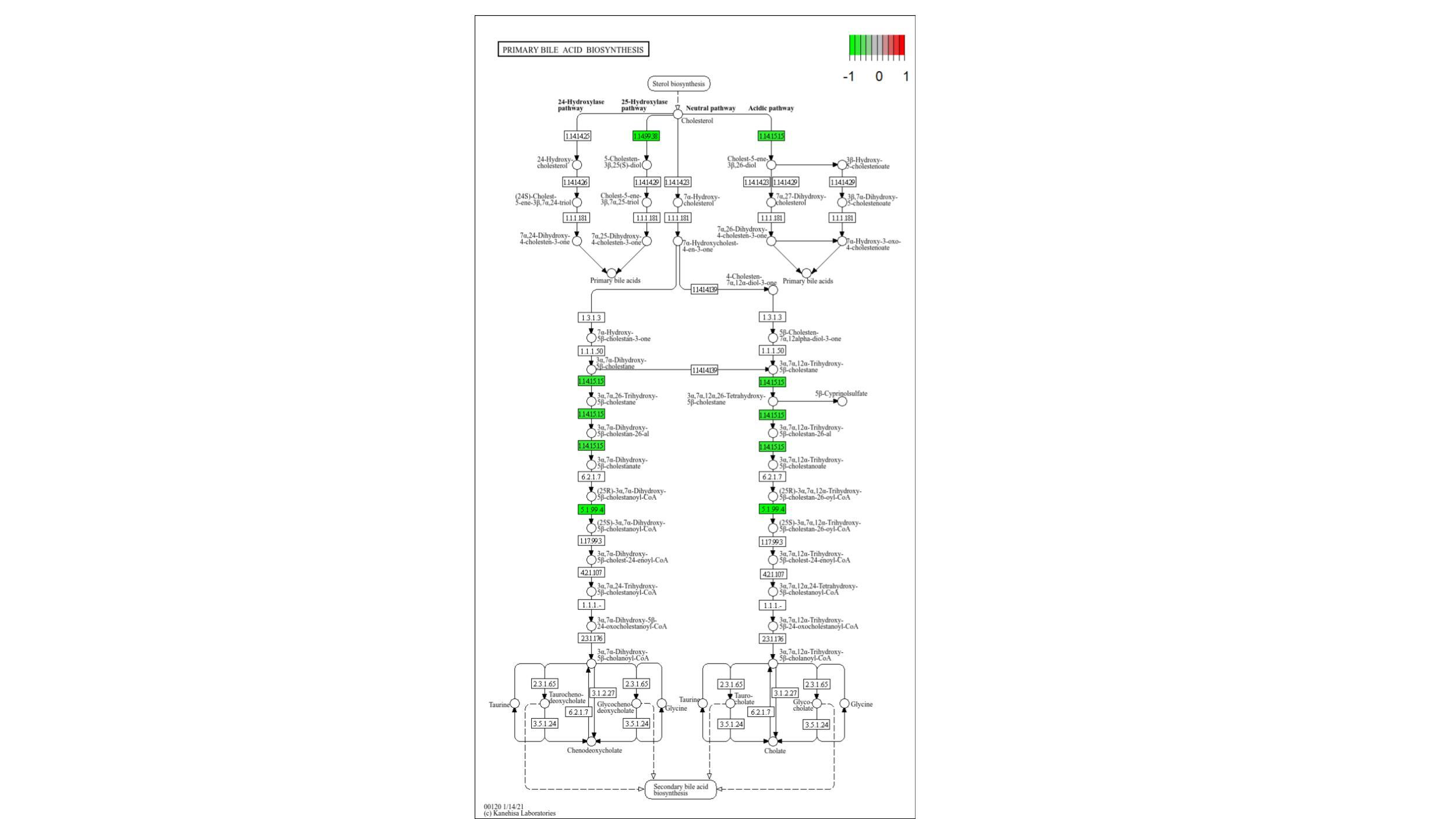

Supplement: Supplementary file 6 [file Image5.jpeg]

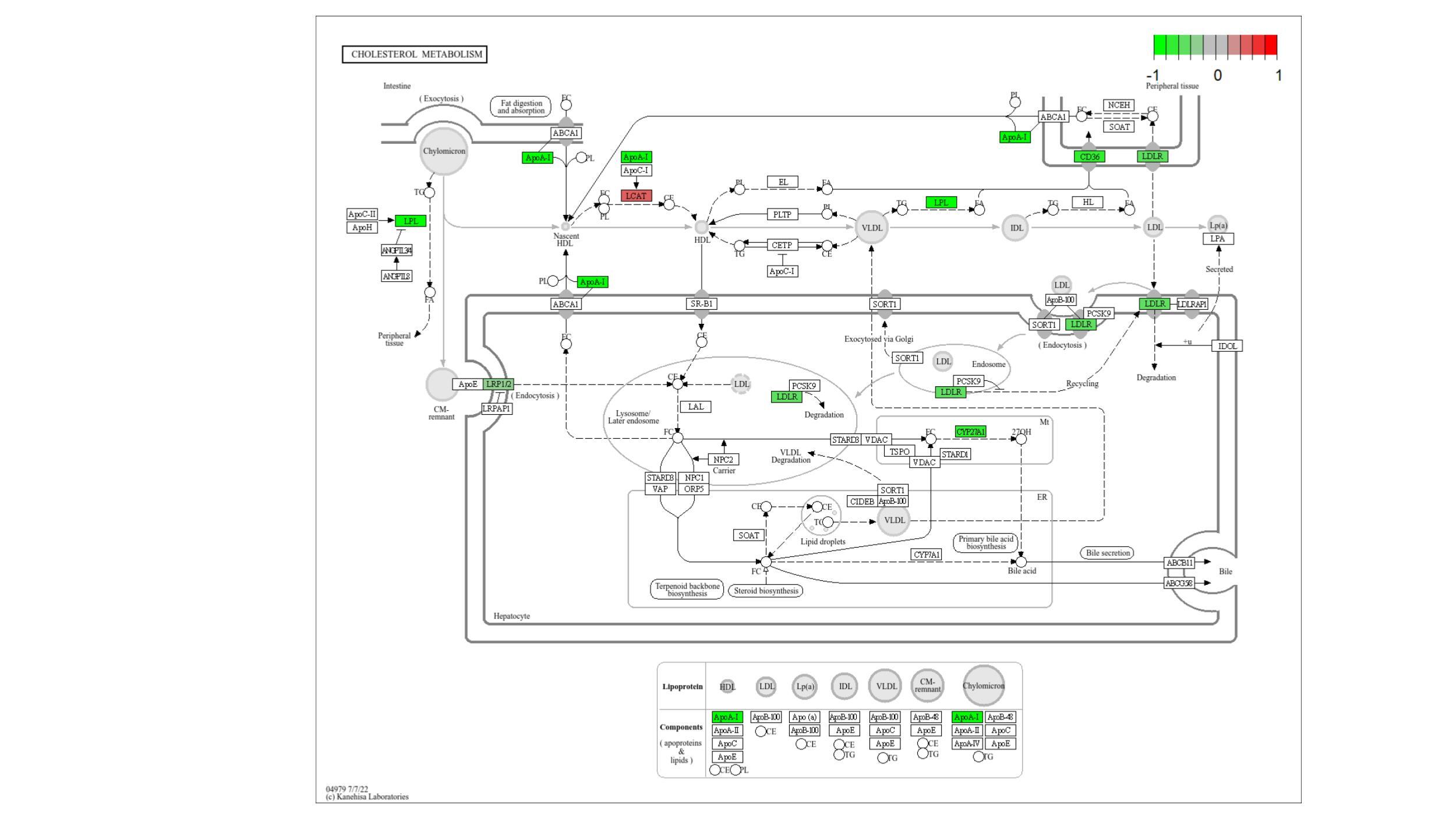

Supplement: Supplementary file 7 [file Image6.jpeg]
